# Maximum rates of climate change are systematically underestimated in the geological record

**DOI:** 10.1038/ncomms9890

**Published:** 2015-11-10

**Authors:** David B. Kemp, Kilian Eichenseer, Wolfgang Kiessling

**Affiliations:** 1 Environment, Earth and Ecosystems, Open University, Walton Hall, Milton Keynes MK7 6AA, UK; 2 GeoZentrum Nordbayern, Fachgruppe PaläoUmwelt, Friedrich-Alexander-Universität Erlangen-Nürnberg, 91054 Erlangen, Germany; 3 Museum für Naturkunde, Leibniz Institute for Research on Evolution and Biodiversity at the Humboldt University Berlin, 10115 Berlin, Germany

## Abstract

Recently observed rates of environmental change are typically much higher than those inferred for the geological past. At the same time, the magnitudes of ancient changes were often substantially greater than those established in recent history. The most pertinent disparity, however, between recent and geological rates is the timespan over which the rates are measured, which typically differ by several orders of magnitude. Here we show that rates of marked temperature changes inferred from proxy data in Earth history scale with measurement timespan as an approximate power law across nearly six orders of magnitude (10^2^ to >10^7^ years). This scaling reveals how climate signals measured in the geological record alias transient variability, even during the most pronounced climatic perturbations of the Phanerozoic. Our findings indicate that the true attainable pace of climate change on timescales of greatest societal relevance is underestimated in geological archives.

## Abstract (web summary)

Recently observed rates of climatic change are typically much higher than those inferred for the geological past. Here, the authors show that maximum rates of climate change inferred from geological data are likely erroneously underestimated.

## Graphical abstract

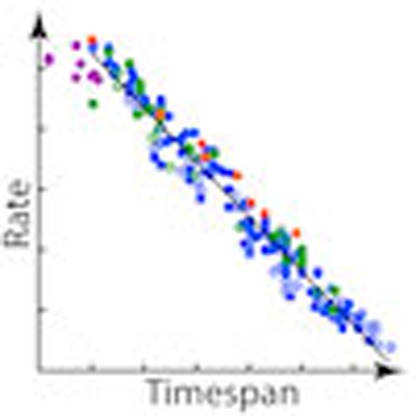

The geological record of past climatic and biological change provides important insights into the responses of the Earth system to large-scale environmental perturbations, and hence the likely responses of the Earth to anthropogenic climate change^1^,^2^. Consequently, extreme events such as the Palaeocene–Eocene thermal maximum (PETM, ∼56 million years ago, Myr ago) and Permian–Triassic boundary (∼252 Myr ago) have been contextualized through comparisons of calculated rates of temperature change to observed and projected modern rates^3^,^4^,^5^. The Permian–Triassic boundary represents the most significant global warming of the Phanerozoic, when Earth's largest mass extinction was accompanied by an increase in tropical sea surface temperature (SST) of ∼15 °C over an interval of nearly 1 Myr (ref. 6). Recent high precision U–Pb dating suggests that ∼10 °C of surface ocean warming occurred over a timespan of ∼60 kyr (ref. 7), that is, a rate of ∼1.7 × 10^−4^ °C per year. In a modern day context, this local rate is ∼42 times lower than the global surface ocean warming of 0.35 °C over the past 50 years^8^. Similarly, through the PETM, a global surface ocean warming of ∼6 °C over 5–20 kyr (refs 3, 4, 9, 10, 11) has been linked to a pronounced perturbation of the global carbon cycle, and suggests a warming rate at least six times slower than modern.

Determining the pace and magnitude of ancient climate change is reliant on the accuracy and validity of palaeotemperature proxies, the accuracy of dating methods and the fidelity of the stratigraphic record for recording climate^9^,^12^. These considerations temper the ability to compare directly ancient and modern rates of change^1^. Nevertheless, the clearest disparity between recent and geological assessments of climate change is the attainable temporal resolution at which changes can be identified and rates determined. A compilation of 194 published oceanic and continental temperature changes spanning the Ordovician period (476 Myr ago) to the present provides a holistic picture of the attainable magnitude and rate of both warming and cooling episodes through Earth history across a range of measurement timespans. We demonstrate that magnitudes and rates of geological temperature changes in this compilation exhibit power law scaling with timespan, emphasising how geological data alias short-term climate variability. Consequently, the true attainable pace of ancient climate change may be commonly underestimated, compromising our understanding of the relative pace (and severity) of both ancient and recent climate change.

## Results

### Compilation

**Figure 1:**
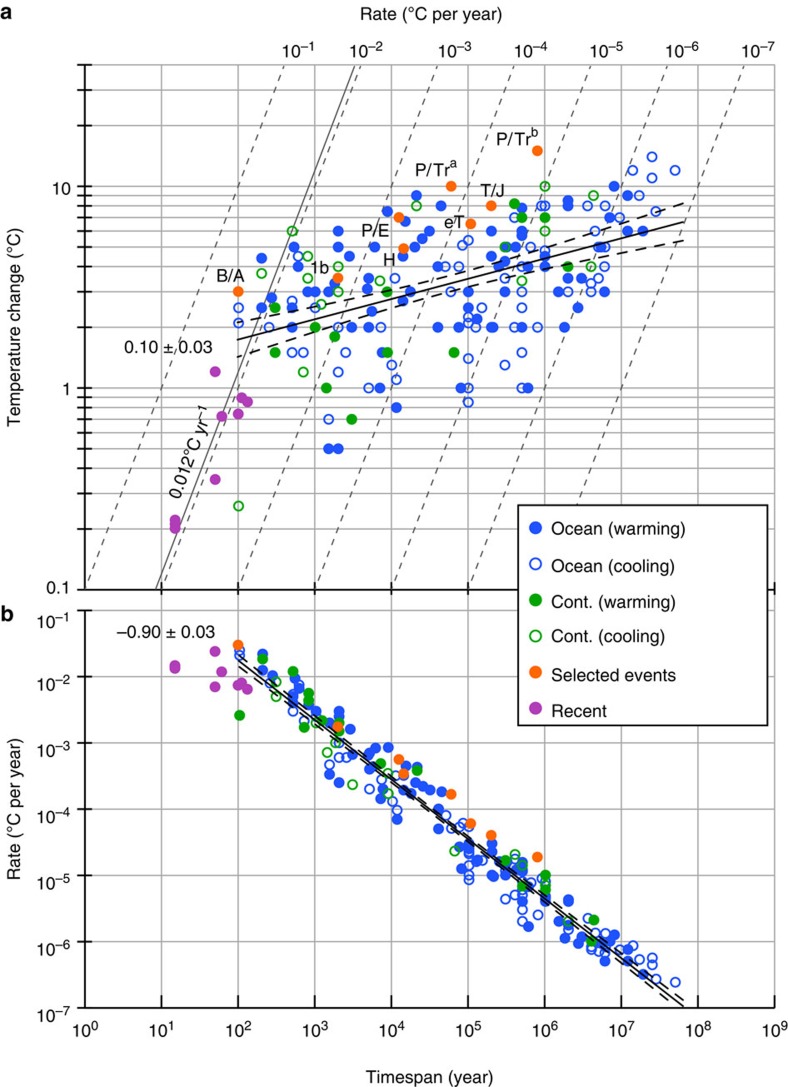
Magnitudes and rates of geological and recent temperature changes plotted against measurement timespan. (**a**) Magnitudes of published oceanic, continental (cont.) and recent temperature changes. Black line is the linear regression slope through the combined ocean and continental geological data (0.10). Dashed lines define the 95% uncertainty envelope of the scaling relationship based on our Monte Carlo error analysis (that is, 0.09±0.03, see main text and Methods). Data associated with well-known warming events are highlighted in orange: B/A=Bølling–Allerød warming (∼13.7 kyr), H=Last Glacial Maximum-Holocene transition (∼11.7 kyr), P/E=Paleocene–Eocene thermal maximum (∼56 Myr ago), 1b=early Albian OAE1b (∼110 Myr ago), eT=early Toarcian (∼182 Myr ago), T/J=Triassic-Jurassic boundary (∼201 Myr ago), P/Tr=Permian–Triassic boundary (∼252 Myr ago). The two points for the P/Tr (a,b) are from refs. 6, 7, respectively, discussed in Introduction. (**b**) Data plotted as rates of temperature change against timespan, highlighting negative power law relationship in continental and oceanic data. The scaling relationship of the combined ocean and continental geological data is –0.90 (black line). As in **a**, dashed black lines define the 95% uncertainty envelope derived from our Monte Carlo error analysis (−0.91±0.03). See Supplementary Data 1 for full data listing.

Our data compilation consists of directly quoted temperature changes representative of the most significant changes in the studied records (Fig. 1, see Methods. Full data listing in Supplementary Data 1). The data underline the near-unprecedented rapidity of recent, directly observed temperature change. Geological rates from individual, localized records only rarely exceeded globally integrated (land and ocean) rates of change determined over the last 150 years^8^,^13^,^14^ (mean ∼0.012 °C per year, Fig. 1). The few geological examples with higher rates are all from the Holocene where measuring timespans of <500 years can be achieved.

### Scaling statistics

Regressed against measurement timespan, the magnitudes of recorded temperature changes scale positively with timespan (*T*, Fig. 1a), while rates of change (*R*) exhibit a negative power law scaling with timespan of the form *R*∼*T*^*a*^ (Fig. 1b). Warming/cooling rates from geological oceanic and continental data exhibit a power law exponent *a* of −0.90±0.03 (2 s.e.) across timespans from 10^2^ to >10^7^ years (Fig. 1b, −0.90±0.03 and −0.85±0.07 in oceanic and continental data, respectively). Data associated with several well-known, large-scale climate system perturbations (including the PETM and Permian–Triassic boundary events) are above the regression line and display a similar scaling trend to the rest of the data (−0.83±0.07, Fig. 1b). Regressing the primary variable of magnitude, rather than rate, against timespan avoids the spurious correlation issue inherent in regression analysis using variables that share a common quantity^15^ (Fig. 1a; see also Methods). This yields a slope of 0.10±0.03 (that is, −0.90+1) as expected, thus confirming the scaling observed from the timespan versus rate analysis (Fig. 1a). Spearman's rank correlation analysis of the relationship between timespan and magnitude in the combined oceanic and continental geological data yields a coefficient of *ρ*=0.47 with a *P* value <1 × 10^−11^ (see Methods, *ρ*=0.45 and *ρ*=0.57, with *P* values of <6 × 10^−9^ and <6 × 10^−4^ in the oceanic and continental data, respectively). For the selected events in Fig. 1a a correlation is also apparent, with *ρ*=0.83 and *P* value=0.016.

### Error modelling

To further assess the robustness of the observed scaling we conducted a Monte Carlo analysis to investigate the effects of uncertainty in the compilation data (see Methods). The key sources of uncertainty are calibration and analytical errors of the proxies, and dating inaccuracies. We generated 10,000 versions of the compilation with randomly applied magnitude errors of ±4 °C (2*σ*) and timespan errors of ±50% (2*σ*). These errors likely exceed the true uncertainty in the magnitude and timespan estimates of the compilation, and hence permit a conservative appraisal of the robustness of the scaling (see Methods). Correlation analysis indicates that *P* values for the relationship between timespan and magnitude are <0.01 in over 99.9% of simulations of the combined ocean and continental data, with the mean slope 0.09±0.03 (2*σ*). The mean slope defining the scaling between timespan and rate in our simulations is −0.91±0.03 (see also Methods). Therefore, reasonable uncertainties in both proxies and timespans are unlikely to affect the scaling relationship we observe.

## Discussion

The scaling relationship predicts that for every 10-fold increase in measurement timespan, there is an approximately 8-fold decrease in the recorded rate of temperature change. The logical explanation for this scaling is that climate change does not proceed in a linear, monotonic manner, but is instead characterized by transient stasis and reversals, even during episodes of extreme warming. Similar explanations have been put forward for observed timespan-dependent scaling in other Earth system processes, notably sedimentation rates^16^ and evolution^17^. Geological temperature changes defined at typically centennial to multimillennial timespans cannot capture the full variance of the climate system operative at shorter timescales^18^,^19^; aliasing variability that is readily apparent from higher resolution and more recent records^20^.

**Figure 2:**
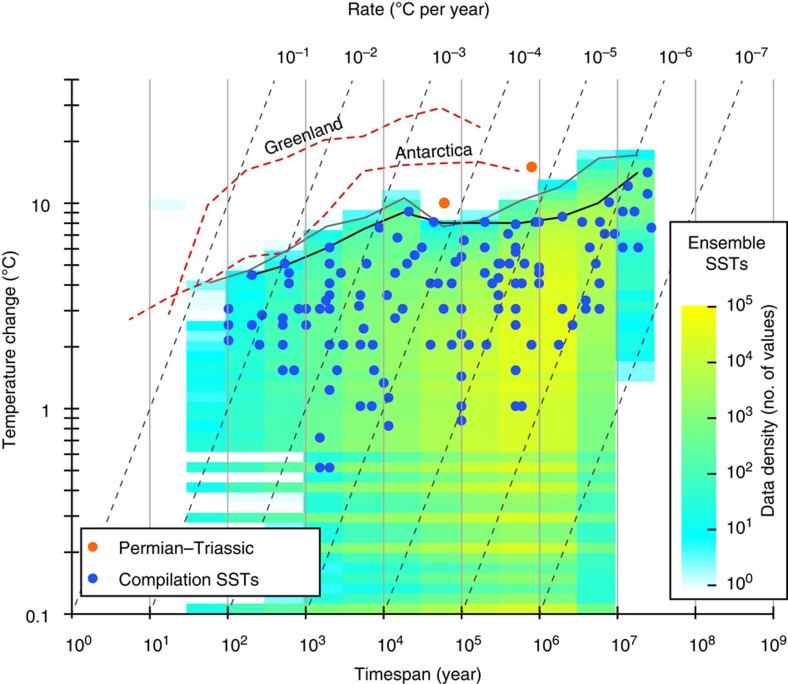
Magnitudes of sea surface and polar temperature changes. Timespan-dependence of sea surface temperature (SST) change is plotted along with maximum magnitudes (heavy black line, see Methods), not including the extreme values of the Permian–Triassic boundary event (orange points). The coloured cluster density plot shows the distribution of >1.6 million temperature changes calculated from an ensemble of 27 single-site SST records, binned into 0.5 × 0.05 log bins (see Methods and Supplementary Table 1). Also shown are maximum magnitudes of temperature changes from the ensemble (grey line) and the Antarctica Dome-C (ref. 24) and Greenland GISP2 (ref. 25) temperature records (red dashed lines).

The validity of this hypothesis and the veracity of the inferred power law is intuitive, based on the demonstrable power law-like behaviour of the climate system on decadal to million year scales^18^,^19^,^21^,^22^ (Fig. 2). Over these timespans, the spectral energy (*S*) of climate scales with frequency (*f*) such that *S*∼*f*^−*β*^, where *β* is the spectral exponent^18^,^19^,^21^,^22^. Systems with spectral power law scaling imply self-affinity and have the property that the expected difference (magnitude) between two points (Δ*x*) scales with time via Δ*x*∼*T*^*h*^, where *h* is the Hausdorff measure^23^. The rate of change versus timespan hence scales as *R*∼*T*^*h*–1^ (that is *a*=*h*–1; ref. 23). The spectral exponent of the system and the Hausdorff measure are related, for 1<*β*<3, by *β*∼2 *h*+1 (ref. 23). Thus, the observed scaling of rates with timespan of ∼−0.90 is the expected product of a climate system with *β*∼1.20. This is within the range previously documented for climatic variability inferred from deep sea, surface ocean and atmospheric proxies (0.8<*β*<2)^18^,^19^,^21^. Our results emphasize that even if the geological record were to record climate perfectly, without bias or error, we should generally observe slower rates of change over longer measurement timespans.

As our compilation is dominated by low- to mid-latitude SST data, we test whether the scaling of SSTs in our compilation is representative of the climate system by comparing our data with the timespan-dependant scaling of more than 1.6 million temperature changes in an ensemble of 27 single-site multiproxy SST records spanning the past ∼13 Myr (Fig. 2; see Methods and Supplementary Table 1). We find a broad match between the two data sets, with maximum magnitudes of SST change in our compilation tracking the maxima attained in the ensembled empirical records, particularly between 10^2^ and ∼10^5^ years (the extreme magnitudes of the Permian–Triassic boundary event notwithstanding, Fig. 2). Between timespans of 10^2^ and ∼10^4^ years, maximum SST magnitudes in our compilation follow closely the scaling of maximum magnitudes in the ensembled SST records (Fig. 2), with *h*∼0.16, implying a *β* of 1.32. This estimate is close to the known average spectral scaling exponent of the climatic continuum inferred from low latitude SST records over the same timespan range (1.29, ref. 19). For comparison, the maximum magnitudes of atmospheric temperature changes from polar latitudes^24^,^25^ (not included in our compilation) have higher values, and exhibit distinct scaling patterns (Fig. 2, see also ref. 19). Within our compilation, data are from latitudes <66°, and the correlation between SST magnitude and latitude/palaeolatitude is not significant (*ρ*=0.14, *P* value=0.11, see also Supplementary Fig. 1a). The scaling is thus not influenced by known latitudinal controls on climate energy distribution^19^.

Taken together, our observations support the inferences that: (1) the data in our compilation are representative of the low to mid-latitude climate system at large; (2) maximum magnitudes/rates of change in our compilation are broadly representative of the attainable limits of this system; and (3) the data and observed scaling are not influenced significantly by biases resulting from compiling data from multiple proxies, locations, ages, background climate states and depositional environments.

Analysis and discussion of geological and recent climate variability has hitherto failed to acknowledge timespan-dependent scaling as a first order control on the observable magnitude and rate of climate change. This has consequences for accurately assessing the impacts of climate change on life. For example, the niche evolution of vertebrates, inferred from ancestor–descendant comparisons over millions of years, has been contrasted with projected rates of climate change in this century to conclude that the rate of warming exceeds the adaptive potential of animals by orders of magnitude^26^. Our work indicates instead that geological episodes of climatic or evolutionary change likely fail to capture the true pace of changes on timescales of most relevance for understanding the impact of similar changes today. Implicitly, our findings also mean that caution must be exercised when describing recent temperature changes as unprecedented in the context of geological rates. If rates of change are to be meaningfully interpreted, then the measurement timespan must be explicitly specified.

**Figure 3:**
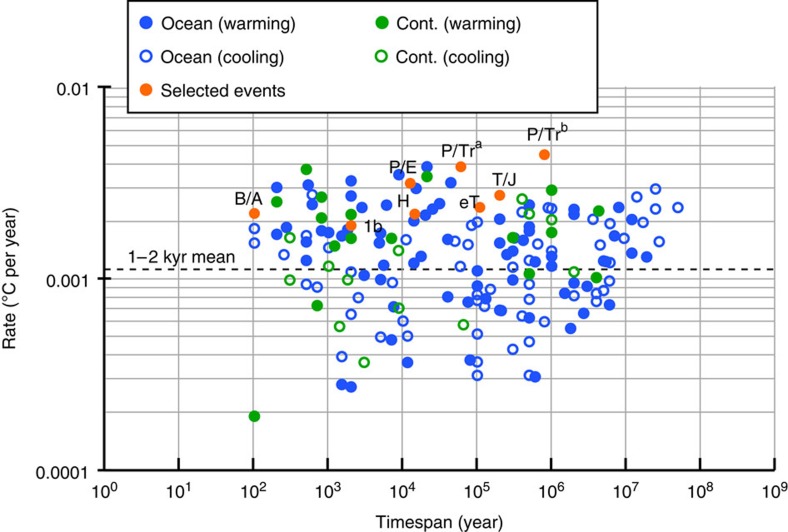
Timespan-corrected climate change rates. Rates were standardized by removing the pervasive timespan-dependent scaling trend in the compilation of continental (cont.) and oceanic data (slope=−0.90; Fig. 1) and normalizing to the mean rate at timespans between 1,000 and 2,000 years (dashed line). This timespan range represents the maximum temporal resolution likely achievable in most palaeoclimate records (see Methods and ref. 12). The normalization emphasizes how the fastest rates of geological climate change were likely attained across the Permian–Triassic boundary event (P/Tr^a^ and P/Tr^b^, ∼252 Myr ago, see also Supplementary Fig. 1b).

Our inferences extend to several of the most pronounced geological climate change events in the Phanerozoic (Fig. 1). Taking into account timespan-dependent scaling, warming rates through intervals such as the Permian–Triassic boundary and the PETM likely exceeded current rates on decadal timescales, at least intermittently (Fig. 3). Warming across the Permian–Triassic boundary stands out as the most significant temperature change of the past ∼0.5 billion years (Figs 2 and 3, see also Supplementary Fig. 1b). The abundance of accompanying evidence for biotic crises and other palaeoenvironmental changes during these extreme events^2^,^6^,^11^,^27^ emphasizes how transient stasis and reversals in long-term temperature trends do not preclude the reality of large-scale climate change with lasting environmental impact, either in the geological past or today.

## Methods

### Data compilation

Our compilation comprises 194 temperature changes and associated timespans taken from 93 publications in the peer reviewed scientific literature (see Supplementary Data 1 for full data set). These changes are quoted in the text of the published works, with timespan data either also quoted or derived from figures or additional references (Supplementary Data 1). The data in the compilation are assumed to be representative of the most relevant climatic changes in a given record and of sufficient significance to the scope of the published work to be worthy of quoting. The temperature changes that our compilation provides are not necessarily representative of global changes. Rather, the data are representative of local palaeoclimatic records, which serve as the only empirical archive of Earth's climatic evolution. SST data dominate the literature and the compilation (132 data points). We did not include polar temperature records owing to the demonstrable higher magnitudes of polar atmospheric temperature change^19^,^22^ (see also Fig. 2). However, 31 terrestrial temperature changes were included to evaluate consistency in the scaling of oceanic and continental data (Fig. 1). Nine data points are of recent temperature changes that have been directly observed over the last <150 years, and are global averages.

Inescapable biases in our compilation include an age-dependence on timespan, because timespans cannot exceed twice the median age of a succession. Equally, timespan is dependent on dating method. Milankovitch dating offers the highest resolving power in records lacking radiocarbon control (that is, any record older than ∼50 kyr) but is likely incapable of providing precision of <1,000 years^12^. Similarly, palaeoproxies have errors typically >1 °C (refs 28, 29, 30). The broad match between the scaling of maximum SST changes in our compilation and the ensembled pattern of SST changes of the past ∼13 Myr (which have age models based primarily on Milankovitch dating) suggest that any biases related to age or dating control do not affect the veracity of our inferences, which is also supported by our Monte Carlo error analysis.

### Ensemble data

Low and mid latitude, multiproxy SST data spanning the past ∼13 Myr were compiled from 27 individual records from 20 separate, globally distributed ocean drilling sites. SST data were compiled using the published proxy temperature calibrations and age models as indicated in Supplementary Table 1.

### Data analysis

Scaling statistics (slopes) were conducted using least squares regression of log-transformed data. Standard error of the slopes in the raw data were calculated using a bootstrap approach (random sampling with replacement) with 10,000 iterations. Hypothesis testing was based on non-parametric correlation tests (Spearman's *ρ*), because all of the timespan data and most of the magnitude data are significantly not normally distributed even after log transformation (Shapiro–Wilk test). The quoted *P* values represent the probability of correlation coefficients at least as high as calculated arising by chance if no correlation existed. As outlined in the main text, our statistical analyses specifically tested the scaling of magnitude with timespan owing to the spurious correlation that arises when investigating the relationship between timespan and rate^15^. Taking the actual timespans but randomly shuffling the associated magnitude data produces a significant correlation (*P* value<0.05) between timespan and calculated rate in 100% of 10,000 trials (mean *ρ* of −0.98). Conversely, 4.84% of trials yielded significant *P* values in the regression analysis of timespan and magnitude (mean *ρ* of 0).

The sensitivity of the scaling relationship between timespan and magnitude of temperature change to errors was tested using a Monte Carlo approach. 10,000 versions of our compilation were created with randomly applied normally distributed timespan errors of ±50% (2*σ*), and normally distributed magnitude errors of ±4 °C (2*σ*). Our compilation is dominated by O-isotope, TEX_86_, alkenone and Mg/Ca proxies (153 data points), which have typical errors of ≤4 °C (refs 1, 28, 29, 30). Our modelled errors likely exceed the magnitude uncertainties in the records we studied because we are interested only in relative changes in temperature within individual records rather than the absolute temperatures. In our approach, magnitude trends can change sign after addition of errors (that is, warming becomes cooling). Tests using a routine that ignored trend reversals caused by the addition of errors (‘rejection sampling') demonstrated that the combined oceanic and continental data have *P* values of <0.01 in a fractionally lower proportion of simulations (99.92% versus 99.99%). Issues of stratigraphic incompleteness are unlikely to impact timespan estimates unless the timespan is defined through a cumulative dating method such as astronomical cycle calibration, where missing cycles lead to an underestimation of true timespan. Errors of the size we use here for Monte Carlo analysis are nevertheless unlikely to result from incompleteness. Indeed, tests using much larger timespan errors of ±70% (2*σ*) still yielded significant *P* values (*P* value<0.01) in >99% of simulations (simulations with timespans that become negative after error addition are rejected). The mean Spearman's *ρ* coefficient describing the correlation between timespan and magnitude in our 10,000 simulations for the combined ocean and continental data is 0.37±0.09 (2*σ*). Analysis of separate continental and ocean data demonstrates that the scaling in the continental data is unlikely to be robust in the presence of significant errors owing to the small size of this data set (*N*=31), with *P* values of <0.05 yielded in ∼76% of simulations, with a mean *ρ* of 0.43±0.22. In contrast, analysis of the oceanic data demonstrates *P* values <0.05 in 99.99% of simulations, with mean *ρ*=0.35±0.10. The mean of the calculated slopes in our simulations was 0.09, with an uncertainty of±0.03 (2*σ*). Thus, the uncertainty in the slope from the Monte Carlo error analysis is the same as that derived from the regression and bootstrapping of the raw data (0.10±0.03), albeit with a fractionally lower slope value. We employed bootstrapping (*N*=1,000) to quantify the 95% uncertainty interval for the calculated slope of each individual Monte Carlo simulation. Our results indicate that slopes were significantly >0 in >99% of the simulations.

To extract timespan-dependent temperature changes from the ensemble and polar temperature data presented in Fig. 2, each temperature value in each individual record was subtracted from every other value in the record to define magnitudes. The published age for each value in the records provided timespan information. The routine worked forwards in time (that is, starting with the oldest value) to define trends (warming or cooling) and avoid duplication. To define maximum attained magnitudes of temperature changes (that is, black and grey lines and red dashed lines in Fig. 2), the magnitude data were binned in 0.5 log bins with the largest value in each bin defining the maximum magnitude for that timespan range.

## Additional information

**How to cite this article:** Kemp, D. B. *et al.* Maximum rates of climate change are systematically underestimated in the geological record. *Nat. Commun.* 6:8890 doi: 10.1038/ncomms9890 (2015).

## Supplementary Material

Supplementary Information Supplementary Figure 1, Supplementary Table 1 and Supplementary References.

Supplementary Data Supplementary Excel file containing all data used in the compilation, and references.
