# Supplementary material for: Maximum rates of climate change are systematically underestimated in the geological record
Source: Nat Commun. 2015 Nov 10;6:8890. doi: 10.1038/ncomms9890 (PMC5227093; doi:10.1038/ncomms9890)
Supplement: Supplementary Information — Supplementary Figure 1, Supplementary Table 1 and Supplementary References. [file ncomms9890-s1.pdf]

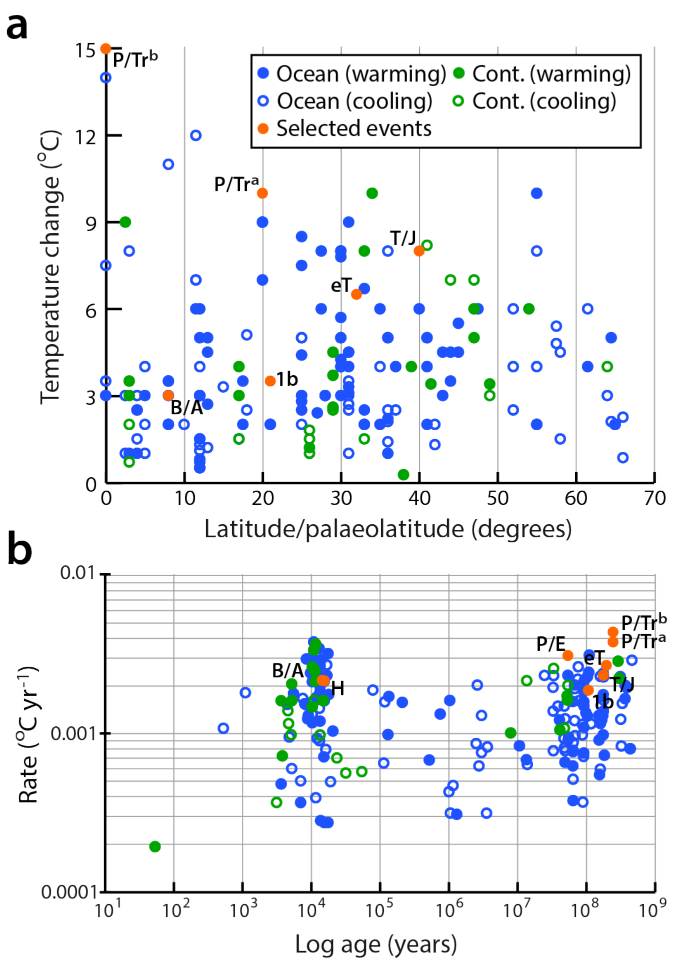


**Supplementary Figure 1. Plots of (palaeo)latitude versus magnitude of geological ocean and continental (cont.) temperature change, and timespan normalised rates versus age**. **a**, Latitudes and palaeolatitudes are taken from the published work containing the data or from related work that references the data or discusses the same localities. Where a palaeolatitude range is provided, the mean value is plotted. No significant correlation exists between latitude/palaeolatitude and magnitude of temperature change (Spearman's $\rho$=0.14, $p$-value=0.07). **b**, Timespan normalised rates are those presented in Fig. 3 of the main text, and are normalised to the mean rate between timespans of 1000 and 2000 years. Note the preponderance of data from the last glacial maximum and Holocene (23-11 kyr), and pre-Oligocene (>34 Ma). B/A = Bølling-Allerød warming (~13.7 kyr), H = Last Glacial Maximum-Holocene transition (~11.7 kyr), P/E = Paleocene-Eocene thermal maximum (~56 Ma), 1b = early Albian OAE1b (~110 Ma), eT = early Toarcian (~182 Ma), T/J = Triassic-Jurassic boundary (~201 Ma), P/Tr = Permian-Triassic boundary (~251 Ma). See main text for further details of these selected events. The Palaeocene-Eocene thermal maximum and Holocene warming events in the compilation are global averages and hence lack (palaeo)latitudes.

**Supplementary Table 1**. **List of records comprising the ensemble SST dataset (Figure 2 of main paper) spanning the past ~13 Myr.**

| Name | SST Proxy | Age from (y) | Age to (y) | Average sampling interval (y) | Latitude (deg.) | Longitude (deg.) | Dataset reference |
|---|---|---|---|---|---|---|---|
| DSDP214 | Mg/Ca (*G. ruber*) | 3552000 | 2013000 | 23677 | 11S | 88E | [1], SSTs recalibrated in [2] |
| | Mg/Ca (*G. sacculifer*) | 4494000 | 3566000 | 29935 | 11S | 88E | [1], SSTs recalibrated in [2] |
| DSDP509 | Mg/Ca (*G. sacculifer*) | 4520000 | 2710000 | 12230 | 0N | 86W | [3], SSTs recalibrated in [2] |
| ODP1010 | UK37 | 13392997 | 38753 | 67107 | 30N | 118W | [4] |
| ODP1021 | UK37 | 13115234 | 40493 | 67050 | 39N | 127W | [4] |
| ODP1143 | Tex86 | 10845310 | 2789210 | 164410 | 9N | 113E | [5] |
| ODP1208 | UK37 | 9914286 | 7453 | 40272 | 36N | 158E | [4] |
| ODP1237 | Mg/Ca (*G. ruber*) | 3690751 | 462661 | 38893 | 16S | 76W | [6], SSTs and age model recalibrated in [2] |
| | Mg/Ca (*G. sacculifer*) | 4371287 | 421459 | 58086 | 16S | 76W | [6], SSTs and age model recalibrated in [2] |
| ODP1239 | UK37 | 3158600 | 496660 | 3568 | 0S | 82W | [7] |
| ODP1241 | Mg/Ca (*G. sacculifer*) | 4518200 | 2433800 | 4561 | 6N | 86W | [8], SSTs recalibrated in [2] |
| ODP658 | Foram. assemblage proxy | 23023 | 88 | 141 | 20N | 18W | [9] |
| ODP709 | Mg/Ca (*G. sacculifer*) | 4514000 | 2192000 | 9251 | 4S | 60E | [10], SSTs recalibrated in [2] |
| ODP763 | Mg/Ca (*G. sacculifer*) | 4512000 | 2004000 | 9393 | 21S | 112E | [10], SSTs recalibrated in [2] |
| ODP806 | Mg/Ca (*G. ruber*) | 470000 | 4300 | 2426 | 0N | 159E | [11] |
| | Mg/Ca (*G. sacculifer*) | 4524000 | 355000 | 9183 | 0N | 159E | [12], SSTs recalibrated in [2] |
| | Tex86 | 12009060 | 95570 | 130917 | 0N | 159E | [5] |
| | UK37 | 5262120 | 95570 | 95677 | 0N | 159E | [5] |
| ODP846 | UK37 | 1833700 | 4000 | 2287 | 3S | 91W | [13] |
| ODP847 | Mg/Ca (*G. sacculifer*) | 4517000 | 392000 | 20122 | 0N | 95W | [12], SSTs recalibrated in [2] |
| ODP850 | Tex86 | 11880530 | 1300 | 148490 | 1N | 110W | [5] |
| | UK37 | 11880530 | 1300 | 148490 | 1N | 110W | [5] |
| ODP982 | UK37 | 4012228 | 0 | 4008 | 57N | 15W | [14] |
| ODP999 | Mg/Ca (*G. ruber*) | 3479764 | 456300 | 188967 | 12N | 78W | [15], SSTs recalibrated in [2] |
| | Mg/Ca (*G. sacculifer*) | 4512685 | 2199722 | 6389 | 12N | 78W | [16], SSTs recalibrated in [2] |
| PL07-39 | Mg/Ca (*G. ruber*) | 24764 | 305 | 133 | 10N | 65W | [17] |
| TR163-19 | Mg/Ca (*G. ruber*) | 361000 | 1000 | 1731 | 2N | 90W | [11] |

## Supplementary references


1. Karas, C. et al. Mid-Pliocene climate change amplified by a switch in Indonesian subsurface throughflow. *Nature Geosci.* **2**, 434-438 (2009).
2. O'brien, C.L. et al. High sea surface temperatures in tropical warm pools during the Pliocene. *Nature Geosci*. **7**, 606-611 (2014).
3. Karas, C., Nürnberg, D., Tiedemann, R. & Garbe-Schönberg, D. Pliocene climate change of the Southwest Pacific and the impact of ocean gateways. *Earth Planet. Sci. Lett.* **301**, 117-124 (2011).
4. LaRiviere, J.P. et al. Late Miocene decoupling of oceanic warmth and atmospheric carbon dioxide forcing. *Nature* **486**, 97-100 (2012).

5. Zhang, Y.G., Pagani, M. & Liu, Z. A 12-million year temperature history of the tropical Pacific Ocean. *Science* **344**, 84-87 (2014).

6. Wara, M.W. & Ravelo, A.C. Data Report: Mg/Ca, Sr/Ca, Mn/Ca, and Oxygen and Carbon Isotope Records of Pliocene–Pleistocene Foraminifers from ODP Leg 202 Site 1237. *Proc. ODP, Sci. Res.* **202** (2006).

7. Etourneau, J., Schneider, R., Blanz, T. & Martinez, P. Intensification of the Walker and Hadley atmospheric circulations during the Pliocene-Pleistocene climate transition. *Earth Planet. Sci. Lett.* **297**, 103-110 (2010).

8. Groeneveld, J. et al. Pliocene development of east-Pacific hydrology as revealed by Mg/Ca analyses on the planktic foraminifer *Globigerinoides sacculifer*. *Proc. ODP, Sci. Res.* **202** (2006).

9. de Menocal, P., Ortiz, J., Guilderson, T. & Sarnthein, M. Coherent high- and low-latitude climate variability during the Holocene Warm Period. *Science* **288**, 2198-2201 (2000).

10. Karas, C., Nürnberg, D., Tiedemann, R. & Garbe-Schönberg, D. Pliocene Indonesian throughflow and Leeuwin current dynamics: implications for Indian Ocean polar heat flux. *Paleoceanography* **26**, doi:10.1029/2010PA001949 (2011).

11. Lea, D., Pak, D. & Spero, H. Climate Impact of Late Quaternary Equatorial Pacific Sea Surface Temperature Variations, *Science* **289**, 1719-1724 (2000).

12. Wara, M.W., Ravelo, A.C. & Delaney, M.L. Permanent El Niño-like conditions during the Pliocene warm period. *Science* **309**, 758-761 (2005).

13. Liu, Z. & Herbert, T.D. High latitude influence on the eastern equatorial Pacific climate in the early Pleistocene epoch. *Nature* **427**, 720-723 (2004).

14. Lawrence, K.T. et al. High-amplitude variations in North Atlantic sea surface temperature during the early Pliocene warm period. *Paleoceanography* **24**, doi:10.1029/2008PA001669 (2009).

15. Seki, O. et al. Alkenone and boron-based Pliocene pCO2 records. *Earth Planet. Sci. Lett.* **292**, 201-211 (2010).

16. Groeneveld, J. *Effect of the Pliocene closure of the Panamanian Gateway on Caribbean and east Pacific sea surface temperatures and salinities by applying combined Mg/Ca and δ18O measurements (5.6-2.2 Ma)*. PhD thesis, University of Kiel (2005).

17. Lea, D.W., Pak, D.K., Peterson, L.C. & Hughen, K.A. Synchroneity of Tropical and High-Latitude Atlantic Temperatures over the Last Glacial Termination. *Science* **301**, 1361-1364 (2003).
